# Supplementary material for: Ion-cluster-mediated ultrafast self-healable ionoconductors for reconfigurable electronics
Source: Nat Commun. 2022 Jun 30;13:3769. doi: 10.1038/s41467-022-31553-4 (PMC9247092; doi:10.1038/s41467-022-31553-4)
Supplement: Supplementary file 1 — Supplementary Information [file 41467_2022_31553_MOESM1_ESM.pdf]

Supplementary Information for

# Ion-Cluster-Mediated Ultrafast Self-Healable Ionoconductors for Reconfigurable Electronics

*Yong Min Kim,[1] Jin Han Kwon,[1] Seonho Kim,[2] U Hyeok Choi,[2] and Hong Chul Moon[1,*]*

[1]Department of Chemical Engineering, University of Seoul, Seoul 02504, Republic of Korea
[2]Department of Polymer Science and Engineering and Program in Environmental and Polymer Engineering, Inha University, Incheon 22212, Republic of Korea

*Corresponding author: H.C.M (hcmoon@uos.ac.kr)

**This PDF file includes:**

Supplementary Table 1 to 3

Supplementary Figure 1 to 26

Supplementary Reference 1 to 29

**Other Supplementary Materials for this manuscript include the following :**

Supplementary Movie 1 to 6

**Supplementary Table 1** Molecular characterizations of polymers prepared in this work.

| Code | $M_w$ (g mol$^{-1}$)[a] | $M_n$ (g mol$^{-1}$)[a] | Polydispersity index ($Đ$)[a] | Mole fraction of hydroxyl parts (%)[b] |
|---|---|---|---|---|
| 100-AAHA | 946,000 | 430,000 | 2.2 | 0 |
| 92-AAHA | 1,274,000 | 490,000 | 2.6 | 8 |
| 61-AAHA | 1,250,600 | 481,000 | 2.6 | 39 |
| 90-DAHA | 898,700 | 473,000 | 1.9 | 10 |

[a]Measured by SEC with poly(ethylene oxide) standards; [b]Determined by $^1$H NMR spectroscopy.

**Supplementary Table 2** Comparison of healing mechanism, efficiency, temperature, and mechanical properties (Young's modulus and fracture strain) of previously reported self-healing platforms, including ionogels, hydrogels, and elastomers.

| **Materials** | **Type** | **Healing Mechanism** | **Healing Efficiency (%)** | **Healing Time (h)** | **Healing Temper-ature (°C)** | **Young's Modulus (MPa)** | **Fracture Strain (%)** | **Ref.** |
|---|---|---|---|---|---|---|---|---|
| PAAm[a]/PNAGA[b] | Hydrogel | hydrogen bond | N.A. | 0.5 | 90 | 0.048 | 665 | 1 |
| UPyHCBA[c] | Hydrogel | hydrophobic interaction, hydrogen bond | N.A. | 0.042 | 25 | 0.0017 | 1000 | 2 |
| PAAc[d]/CTA[e] | Hydrogel | hydrophobic interaction | 75 | 0.5 | 80 | 0.016 | 900 | 3 |
| MPTC[f]/NaSS[g]/DMAEA[h]/BIS[i] copolymer | Hydrogel | electrostatic interaction | 75 | 0.125 | 25 | 0.036 | 650[a] | 4 |
| P(NaSS-*co*-DMAEA-Q) | Hydrogel | electrostatic interaction | 82.3 | 24 | 25 | 0.08 | 1000 | 5 |
| Zwitterionic nanocomposite hydrogel | Hydrogel | electrostatic interaction | 74 | 24 | 25 | 0.0046[x] | 2000 | 6 |
| Agel[j]–DCMC[k] hydrogel | Hydrogel | imine bond | 90 | 1 | 37 | 0.03 | 140 | 7 |
| P(AM-*co*-AEAM[l]) hydrogel | Hydrogel | hydrogen bond, ionic interaction, Schiff base bond | 53 | 48 | 25 | 0.02[x] | 1205 | 8 |
| P(Urea-IL[m]-SPMA[n]) | Hydrogel | electrostatic interaction, hydrogen bond | 91 | 24 | 25 | 0.18 | 720 | 9 |
| hyperbranched polyurethane (HPDU) | Elastomer | hydrogen bond | 96.5 | 10 | 90 | 0.8[x] | 3100 | 10 |
| Dopamine acrylamide/butyl acrylate/$Ca^{2+}$ | Elastomer | hydrogen bond, metal coordination | 37 | 24 | 25 | 3.9 | 310 | 11 |
| poly(AAm/ChCl[o]-co-MA[p]/ChCl) | Elastomer | hydrogen bond | 91 | 72 | 20 | 0.1 | 450 | 12 |
| polyurethane acrylate | Elastomer | disulfide bond hydrogen bond | 95 | 12 | 80 | 0.5[x] | 400 | 13 |
| polyurethane (DA-PU) | Elastomer | hydrogen bond, donor-acceptor interaction | 97 | 6.67 | 60 | 1.33[x] | 1900 | 14 |
| PU-HEDS[q]-400 | Elastomer | hydrogen bond, disulfide bond | 95.4 | 24 | 50 | 5.04 | 553 | 15 |
| Fe-Hpdca[r]-PDMS | Elastomer | metal-ligand interaction | 90.3 | 48 | 25 | 0.54 | 1700 | 16 |
| cellulose nanocrystal/ [BMIM][$HSO_4$] composite | Ion gel | electrostatic interaction | 90.3 | 24 | 25 | 0.016 | 620 | 17 |
| PS[s]-*b*-P(DMAAm[t]-*r*-AAc)/[EMI][TFSI] | Ion gel | hydrogen bond | 90 | 3 | 25 | 0.5[x] | 400 | 18 |
| TetraPEG-Ant[u] /[EMI][TFSI] | Ion gel | photoisomeriza-tion | 66 | 10 | 150 | 0.007[x] | 950 | 19 |
| P(AzoMA-*r*-NIPAm)-*b*-PEO-*b*-P(AzoMA-*r*-NIPAm)[v]/[BMI][$PF_6$] | Ion gel | photoisomeriza-tion | 81 | 64 | 36 | 0.02[x] | 450 | 20 |

| | | | | | | | | |
|---|---|---|---|---|---|---|---|---|
| PVDF-*co*-HFP[w]-5545/[EMI][OTf] | Ion gel | ion-dipole interaction | 100.3 | 6 | 50 | 0.10 | 1500 | 21 |
| PVDF-*co*-HFP-5545/[EMI][TFSI] | Ion gel | ion-dipole interaction | 99.1 | 24 | 50 | 0.21 | 2000 | 22 |
| Cellulose/[BMI][Cl] | Ion gel | hydrogen bond | 96 | 2 | 25 | 1[x] | 60 | 23 |
| **92-AAHA-IL** | **Ion gel** | **ion cluster** | **90.3**<br>**98.7**<br>**99.7** | **0.016 (1min)**<br>**0.050 (3min)**<br>**0.083 (5min)** | **25** | **0.84** | **1130** | **This work** |

[a]PAAm: poly(acrylamide), [b]PNAGA: poly(*N*-acryloyl glycinamide), [c]UPyHCBA: 4-(6-(3-(6-methyl-4-oxo-1,4-dihydropyrimidin-2-yl)ureido)hexylcarbamoyloxy)butyl acrylate, [d]PAAc: poly(acrylic acid), [e]CTA: cetyltrimethylammonium, [f]MPTC: [3-(methacryloylamino)propyl]trimethylammonium chloride, [g]NaSS: sodium p-styrenesulfonate hydrate, [h]DMAEA: 2-(dimethylamino)-ethyl methacrylate, [i]BIS: N,N′-methylenebis(acrylamide), [j]Agel: amino-gelatin, [k]DCMC: dialdehyde carboxymethyl cellulose, [l]AEAM: 2-aminoethyl acrylamide hydrochloride, [m]Urea-IL: imidazolium-based ionic liquid (IL) monomers with urea groups, [n]SPMA: 3-sulfopropyl methacrylate potassium salt, [o]ChCl: choline chloride, [p]MA: maleic acid, [q]HEDS: 2,2‘-hydroxy ethyldisulfide, [r]Hpdca: 2,6-pyridinedicarboxamide, [s]PS: poly(styrene), [t]DMAAm: N,N-dimethylacrylamide, [u]TetraPEG-Ant: tetra-arm poly(ethylene glycol)-anthracene, [v]P(AzoMA-*r*-NIPAm)-*b*-PEO-*b*-P(AzoMA-*r*-NIPAm): Poly(4-phenylazophenyl methacrylate-*random*-N-isopropylacrylamide)-*block*-poly(ethylene oxide)-*block*-P(4-phenylazophenyl methacrylate-*random*-N-isopropylacrylamide),[w]PVDF-*co*-HFP: Poly(vinylidene fluoride-*co*-hexafluoropropylene).[x]calculated from produced stress-strain curve.

**Supplementary Table 3** Vogel-Fulcher-Tammann (VFT) temperature fitting parameters of temperature dependence of the $\alpha_2$ process.

| Sample | $\log(\omega_\infty)$ (rad s$^{-1}$) | $D$ | $T_0$ (K) | $T_g$[a] (K) | $E_a$ (kJ/mol) |
|---|---|---|---|---|---|
| 92-AAHA-IL | 10.6 | 6 | 171 | 256 | 9 |
| 90-DAHA-IL | 12.6 | 11 | 165 | 250 | 15 |

[a]Obtained from DSC thermograms (Supplementary Fig. 7).

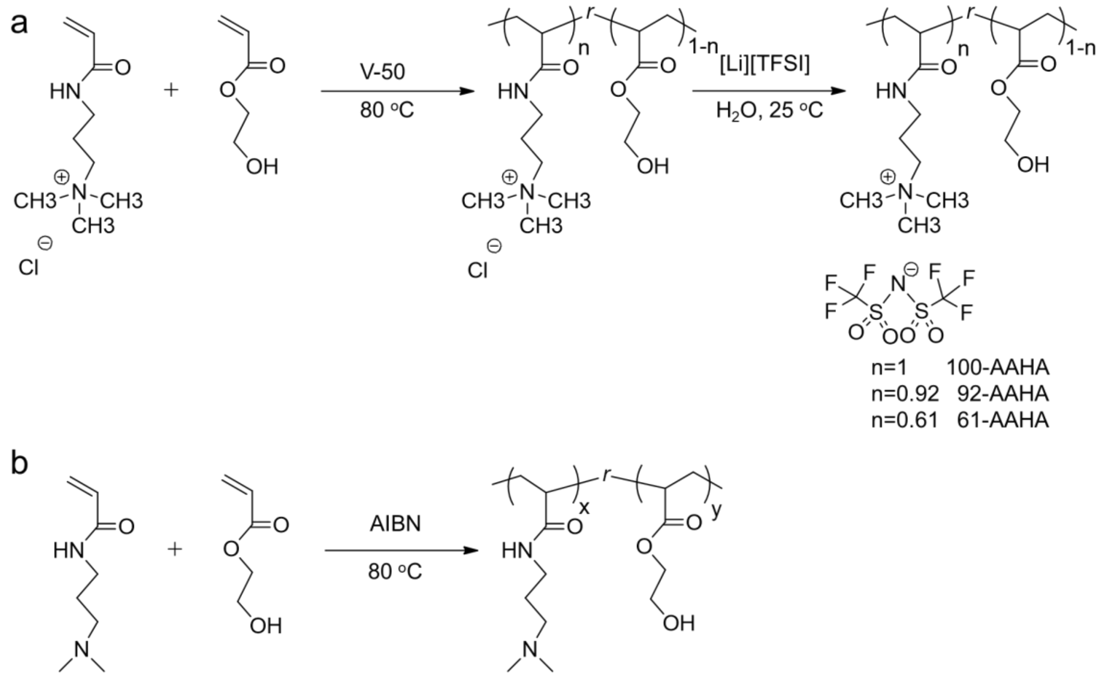


**Supplementary Fig. 1 Synthetic strategy for copolymers employed in this work.** Synthetic routes for preparing **a** AAHAs and **b** DAHA.

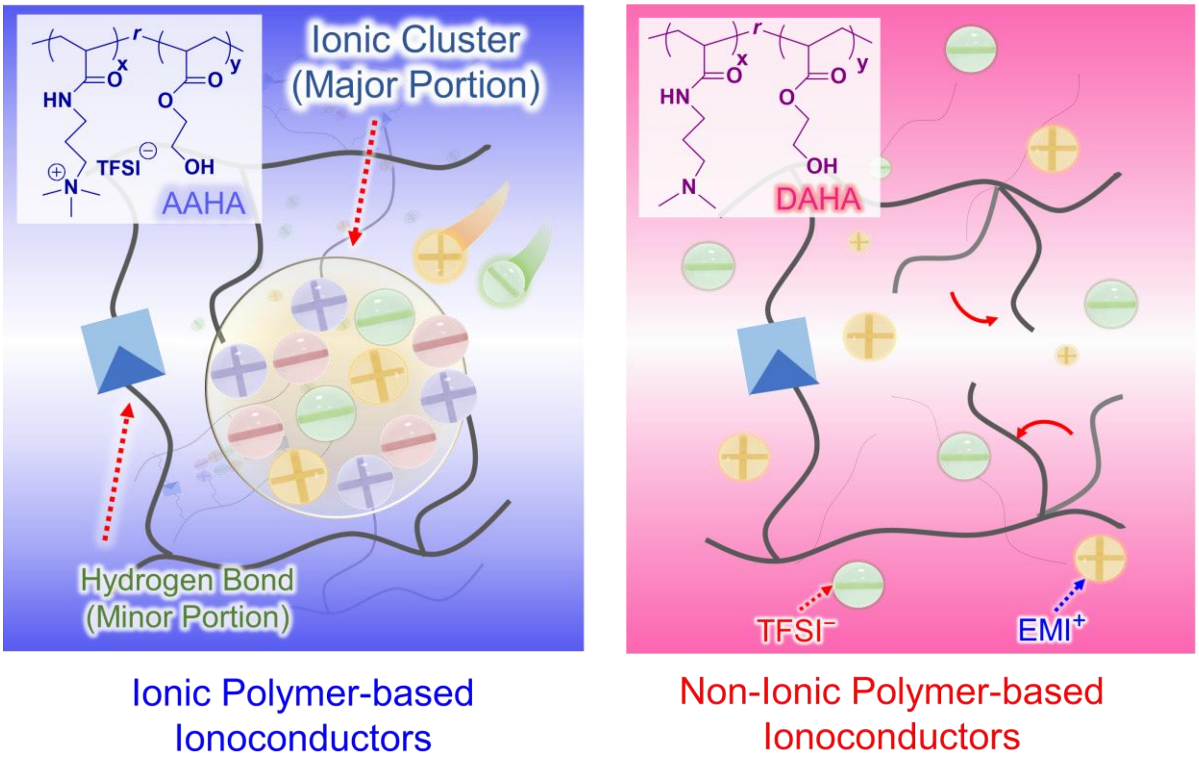


**Supplementary Fig. 2 Difference in molecular interaction depending on the presence of ionic moiety.** Schematic illustrations of ionic polymer-based ionoconductor (92-AAHA-IL), in which both hydrogen bonding and ion-cluster formation take place, and non-ionic polymer-based ionoconductors (90-DAHA-IL) only including hydrogen bonding interactions.

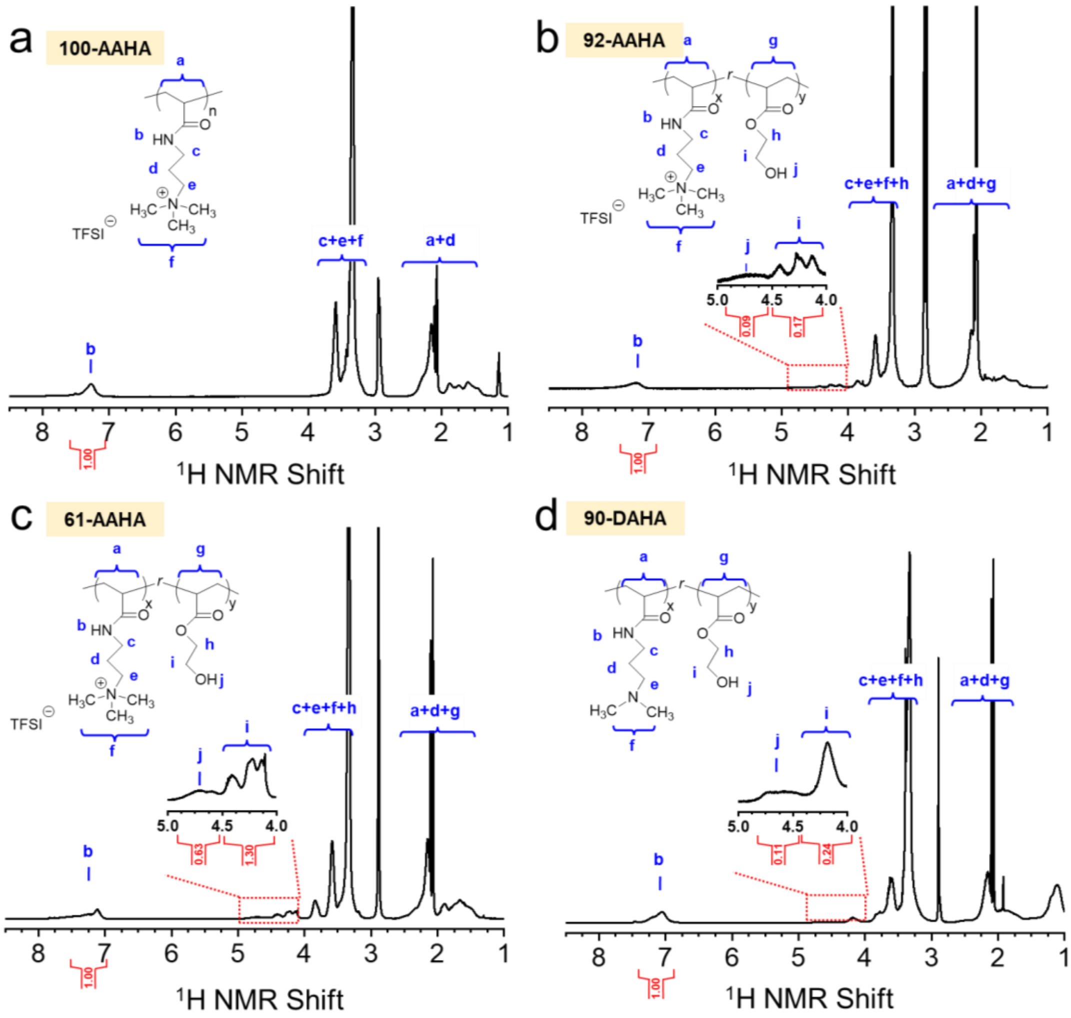


**Supplementary Fig. 3 Structural analysis of used polymers.** $^1$H NMR spectra of prepared polymers. **a** 100-AAHA, **b** 92-AAHA, **c** 61-AAHA, and **d** 90-DAHA.

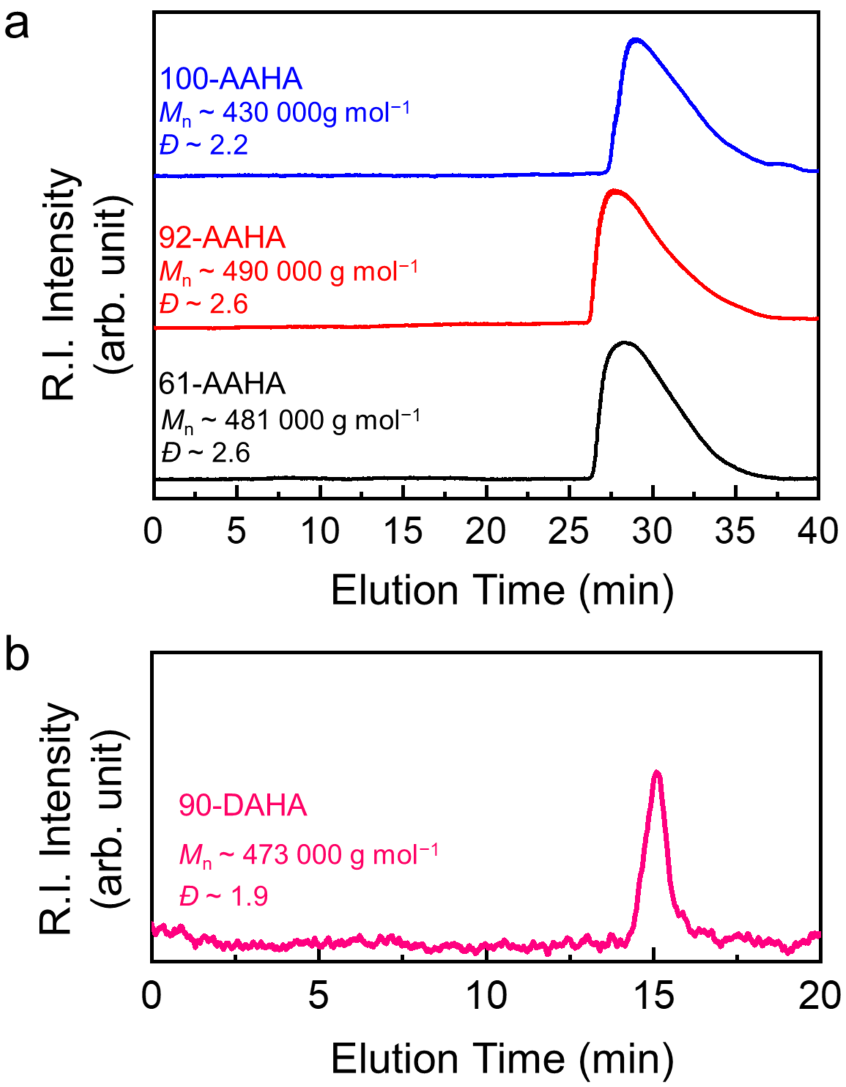


**Supplementary Fig. 4 Molecular characteristics of used polymers.** SEC traces of polymers employed in this study. **a** 100-AAHA, 92-AAHA, and 61-AAHA. To prepare the GPC samples, the synthesized AAHAs were dissolved in an aqueous buffer (pH = 3) containing 0.1 M sodium phosphate, and the operating flow rate was fixed at 0.5 mL $min^{-1}$. **b** SEC trace of 90-DAHA dissolved in HPLC water with a flow rate of 1.0 mL $min^{-1}$. All samples were calibrated with poly(ethylene oxide) standards.

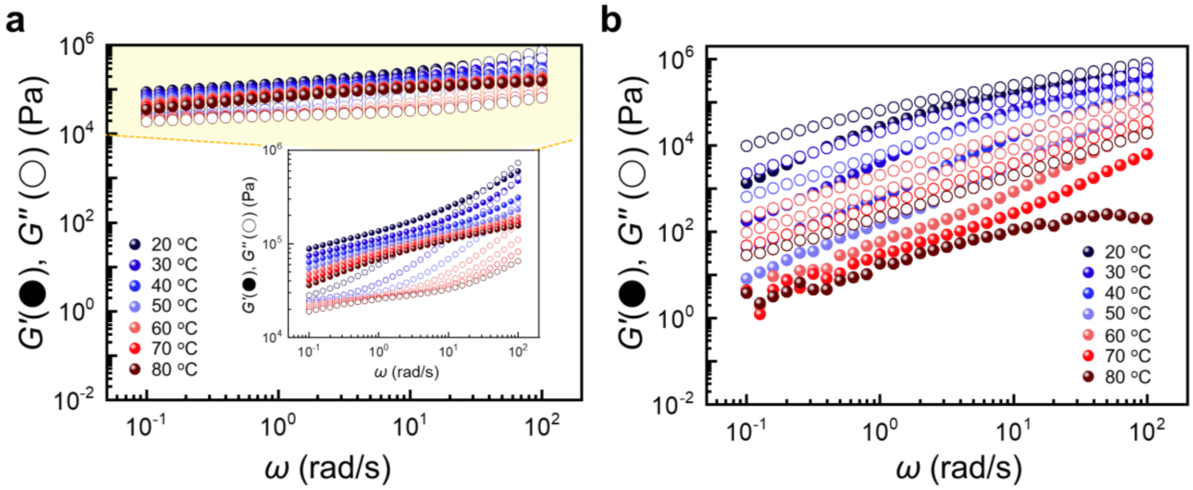


**Supplementary Fig. 5 Difference in rheological behaviors.** Frequency-dependent dynamic storage (filled circle) and loss (open circle) moduli at various temperatures for **a** 92-AAHA-IL and **b** 90-DAHA-IL.

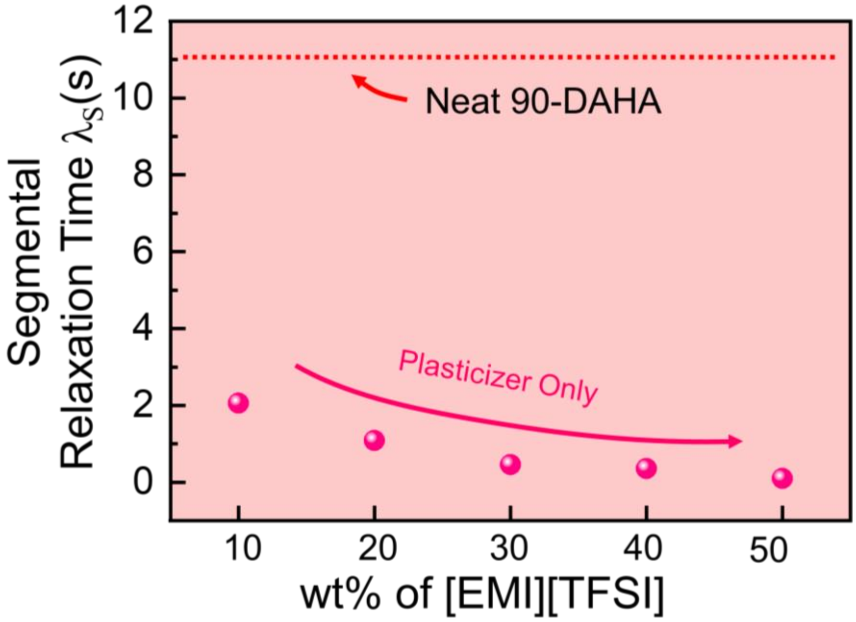


**Supplementary Fig. 6 Effect of [EMI][TFSI] on non-ionic polymer-based system.** Changes in segmental relaxation time of 90-DAHA-IL according to the weight fraction of the introduced [EMI][TFSI]. The red dotted-line indicates the segmental relaxation time of neat 90-DAHA.

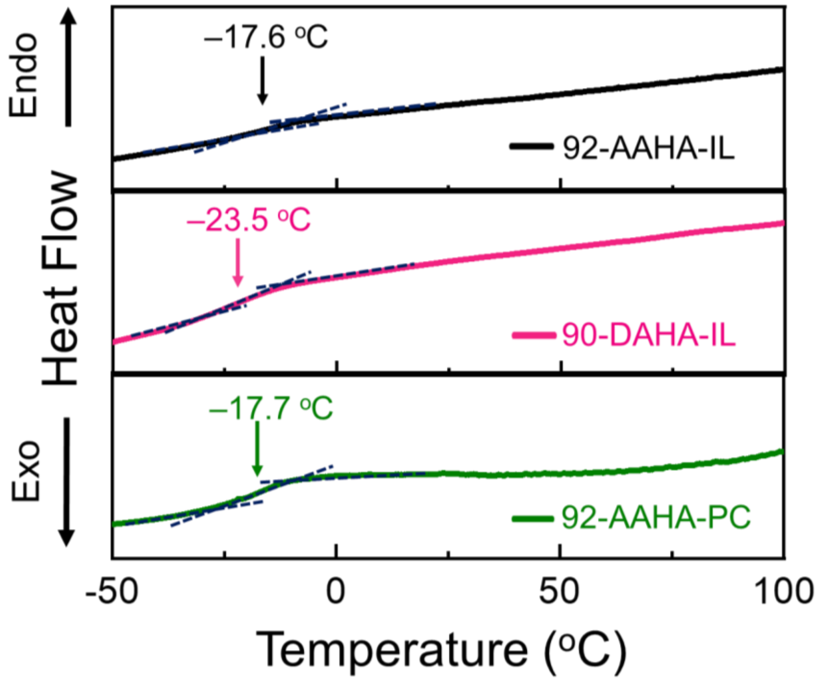


**Supplementary Fig. 7 Thermal properties of various ionoconductors with different copolymers.** DSC thermograms of 92-AAHA-IL, 90-DAHA-IL, and 92-AAHA-PC, obtained during the second run at a heating rate of 10 ºC min$^{-1}$.

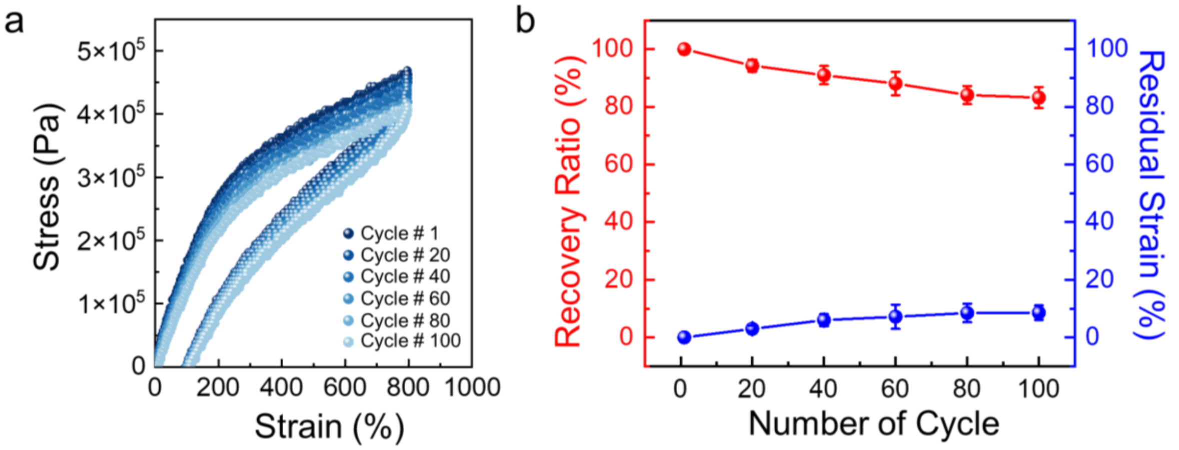


**Supplementary Fig. 8 Mechanical stability of 92-AAHA-IL. a** Stress-strain curves during cyclic stretching/releasing at 800% and **b** corresponding changes in recovery ratio and residual strain. The recovery ratio of ~83.2% and residual strain of ~8.6% after 100 consecutive cycles represent the high mechanical reliability of 92-AAHA-IL. Error bars indicate standard deviation.

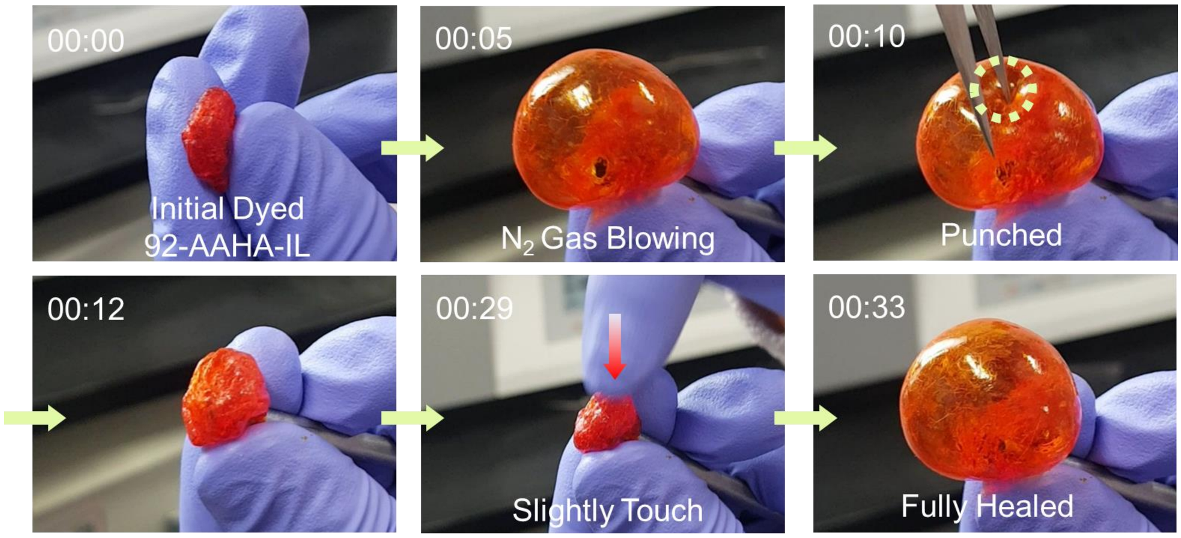


**Supplementary Fig. 9 Demonstration of ultrafast self-healing performance of 92-AAHA-IL.** Snapshots from Supplementary Movie 1 showing the immediate self-healing capability of 92-AAHA-IL. The balloon-shaped 92-AAHA-IL was expanded by blowing $N_2$ gas and punched with a tweezer to induce mechanical damage. After self-healing with a slight touch to contact the injured region, 92-AAHA-IL fully prevented the leakage of $N_2$ molecules.

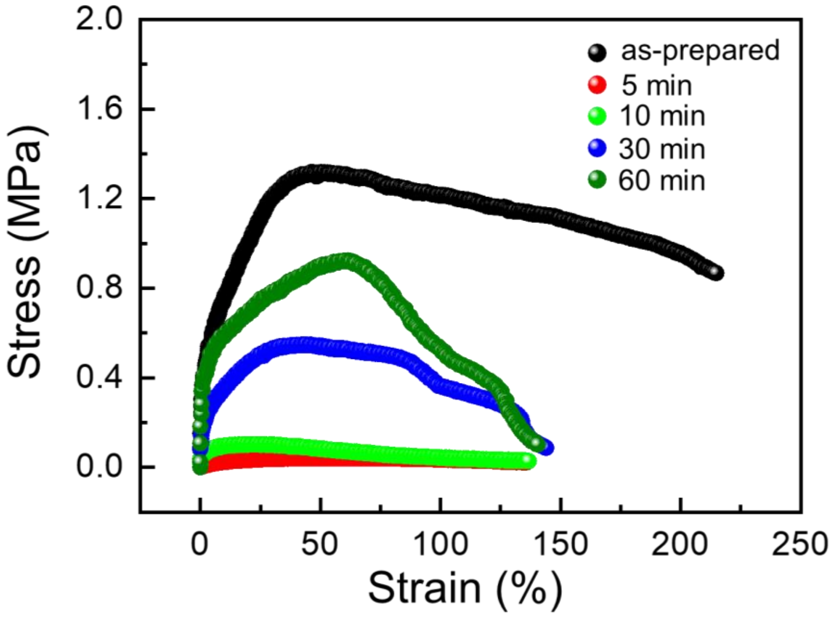


**Supplementary Fig. 10 Correlation between healing time and mechanical properties of 90-DAHA-IL.** Tensile stress-strain curves of as-prepared and healed 90-DAHA-IL (5−60 mins) at 25$^o$C.

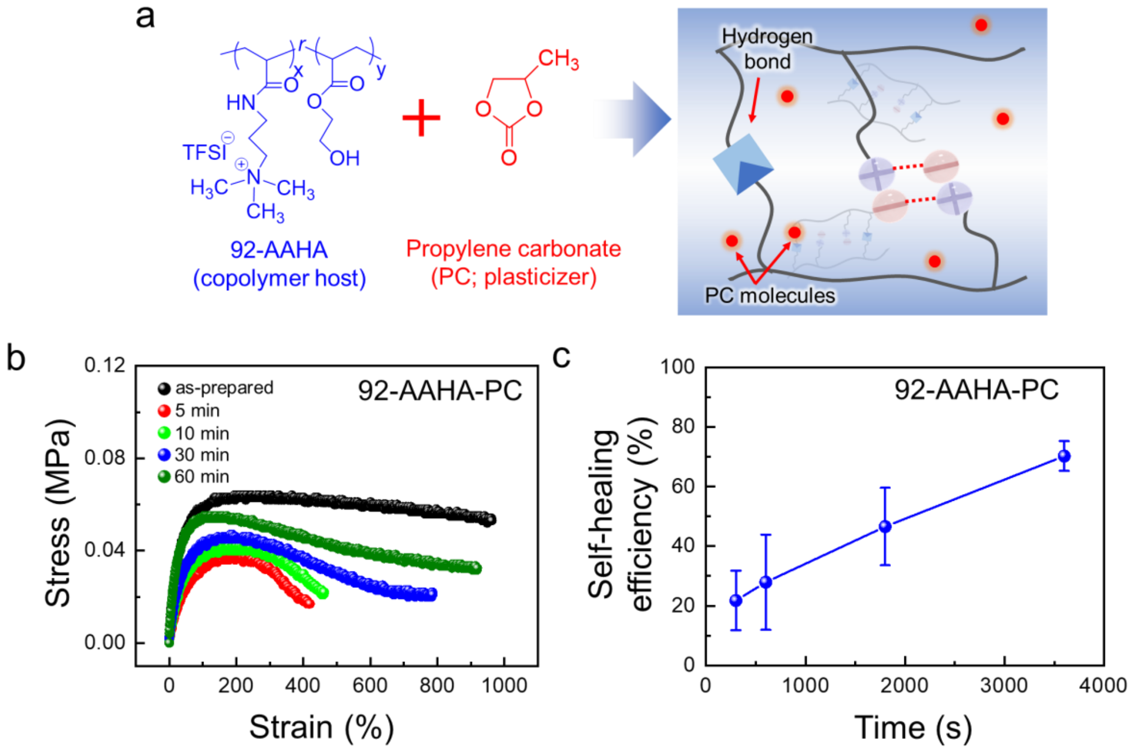


**Supplementary Fig. 11 Mechanical and self-healing characteristics of the system with no ionic liquids. a** Schematic illustration of 92-AAHA-PC. **b** Tensile stress–strain curves for self-healing tests of 92-AAHA-PC at 25 °C, and **c** self-healing efficiency as a function of healing time. The self-healing efficiency was calculated from the ratio of the toughness values before damage and after healing obtained from the stress–strain curves. Error bars indicate standard deviation.

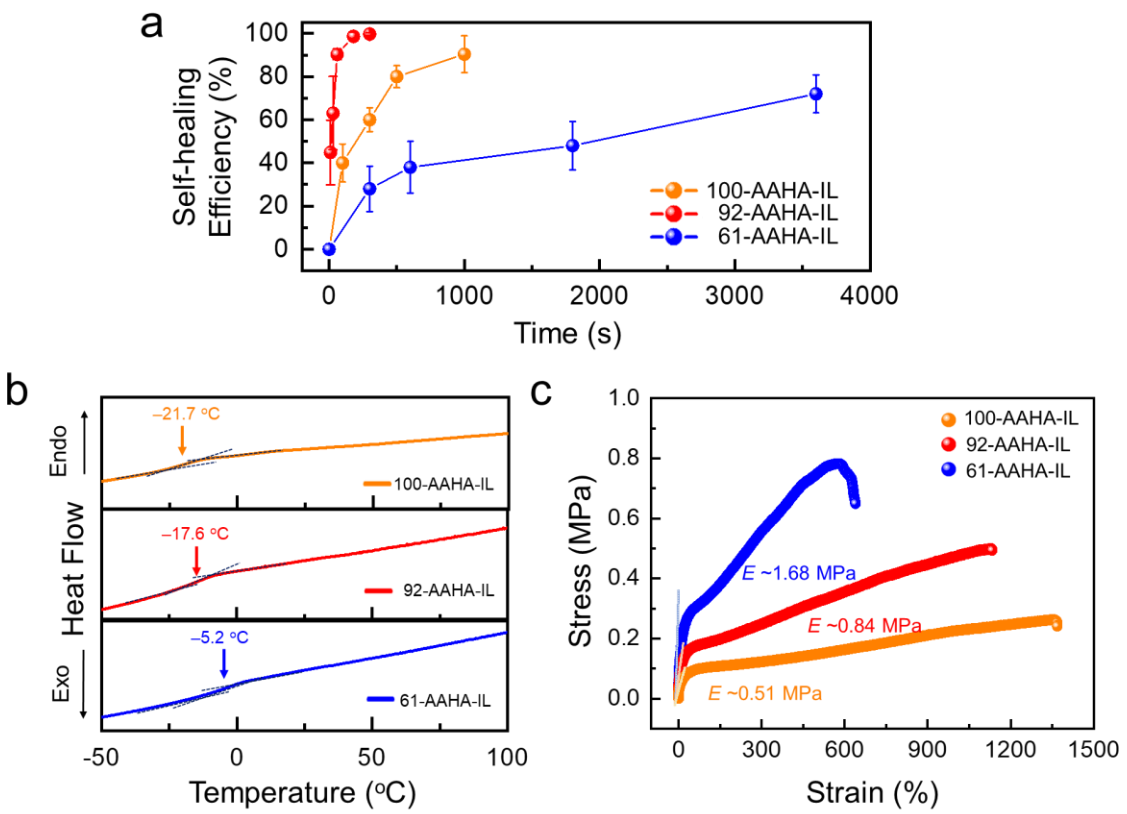


**Supplementary Fig. 12 Dependence of self-healing, thermal, and mechanical performance on the ionic content in AAHA-IL.** **a** Differences in self-healing efficiencies measured with different copolymer hosts. Error bars indicate standard deviation. **b** DSC thermograms for evaluating $T_g$ values. **c** Tensile stress–strain curves for determining elastic moduli of 100-AAHA-IL, 92-AAHA-IL, and 61-AAHA-IL.

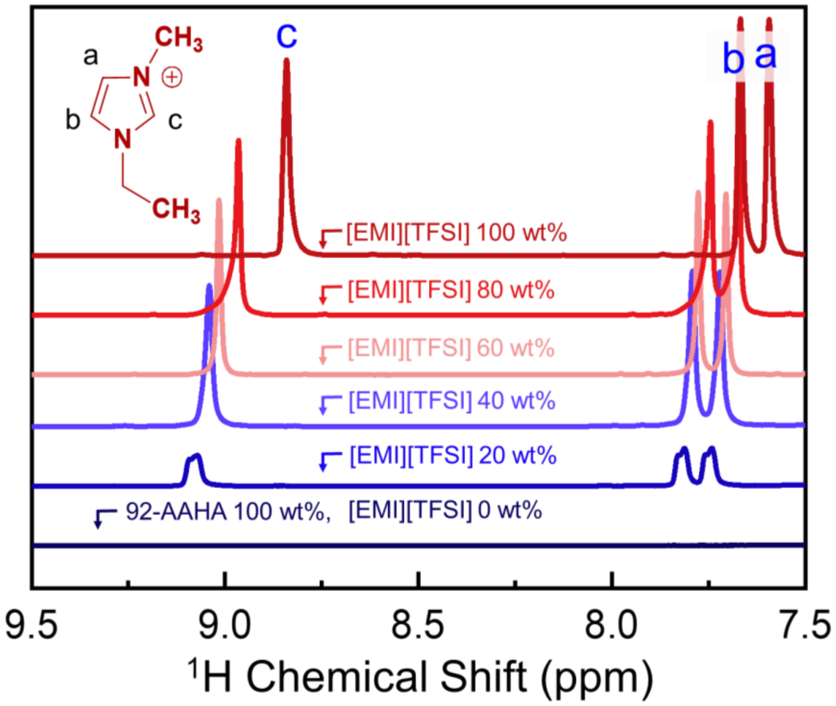


**Supplementary Fig. 13 Interaction between [EMI][TFSI] and 92-AAHA.** $^1$H NMR spectra of [EMI][TFSI] with various 92-AAHA-IL contents. The contents of [EMI][TFSI] were 0 (navy line, neat 90-DAHA), 20, 40, 60, 80, and 100 wt.% (red line, neat [EMI][TFSI]). The representative $[EMI]^+$ peaks were slightly shifted when the 92-AAHA was added, but no peaks below 7.5–9.5 ppm were observed for neat 92-AAHA.

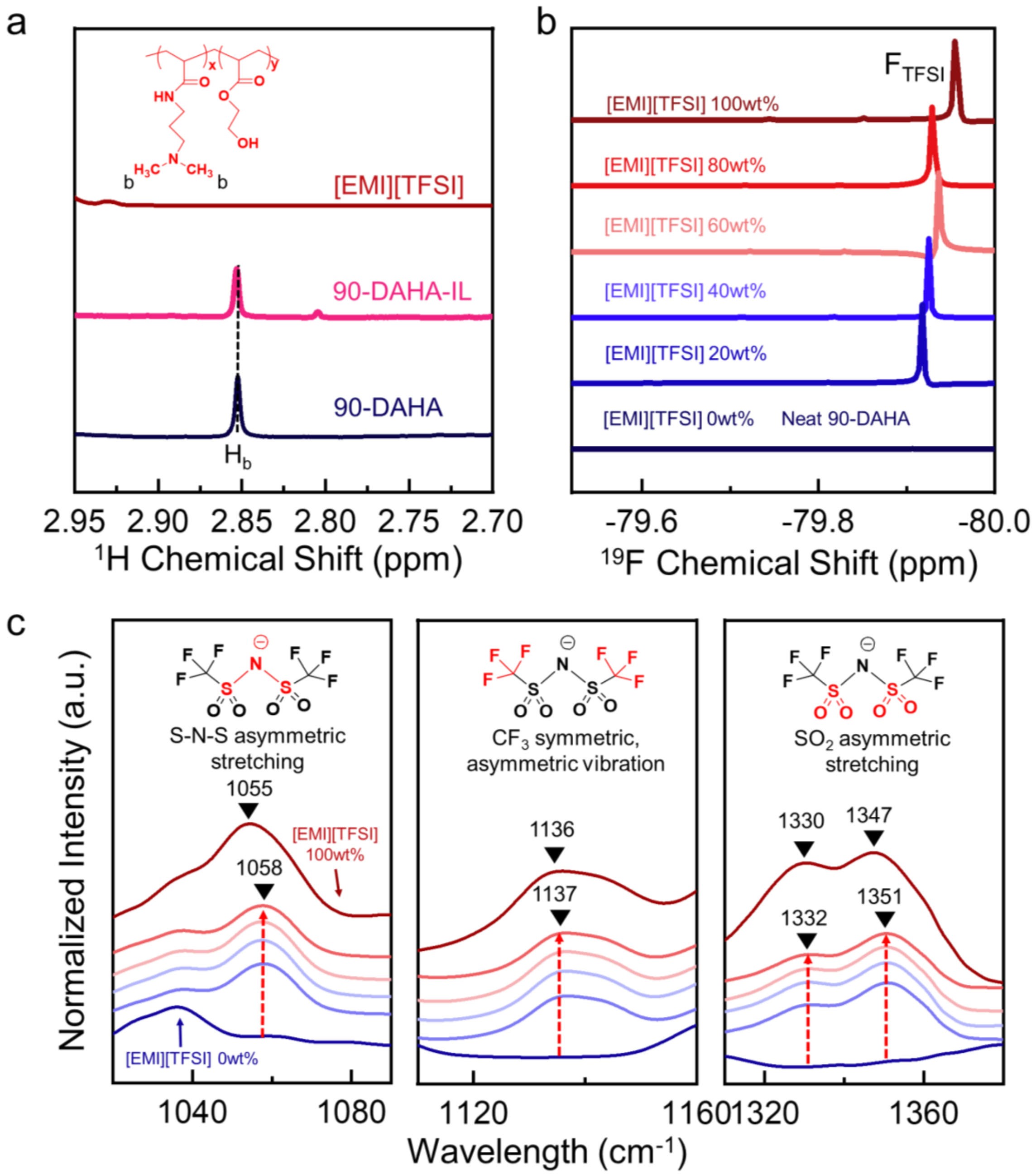


**Supplementary Fig. 14 Spectroscopic analysis of 90-DAHA-containing system. a** Comparison of $^1$H NMR spectra of 90-DAHA, 90-DAHA-IL, and [EMI][TFSI]. 90-DAHA-IL contained 30 wt.% [EMI][TFSI]. **b** $^{19}$F NMR spectra of 90-DAHA-IL with various [EMI][TFSI] contents. No [TFSI]$^-$ peak was observed for neat 90-DAHA. **c** Changes in FTIR absorption peaks with respect to the fraction of [TFSI]$^-$ in 90-DAHA-IL. The contents of [EMI][TFSI] were 0 (navy line, neat 90-DAHA), 20, 40, 60, 80, and 100 wt.% (red line, neat [EMI][TFSI]).

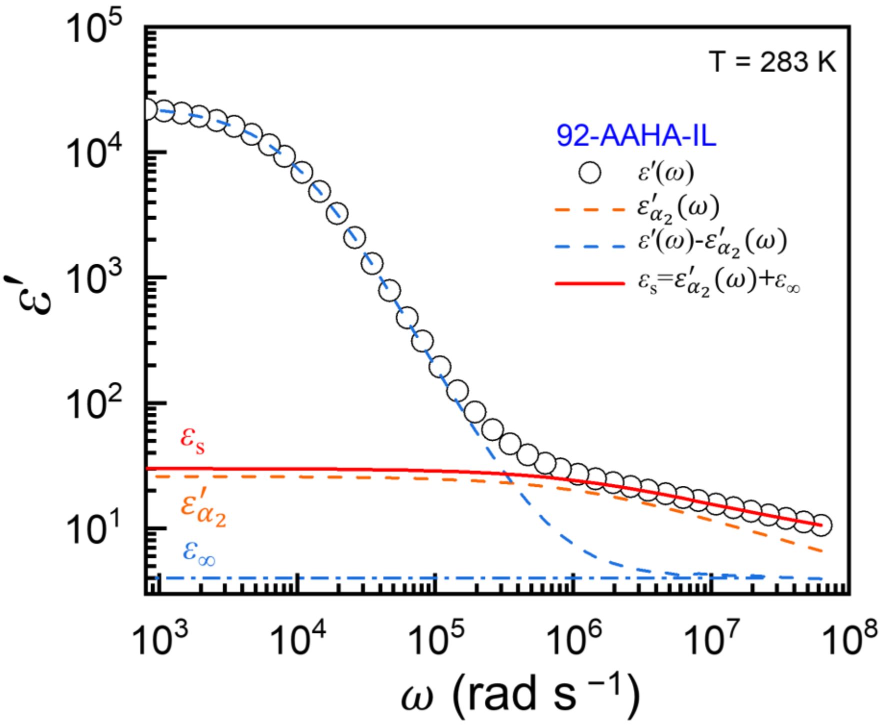


**Supplementary Fig. 15 Frequency dependent dielectric characteristics of 92-AAHA-IL.** Angular frequency dependence of dielectric permittivity $\varepsilon'(\omega)$ of 92-AAHA-IL at 283 K and corresponding static dielectric constant $\varepsilon_s$ (red solid line), determined as the sum of the frequency-dependent contributions from the $\alpha_2$ process $\varepsilon'_{\alpha_2}(\omega)$ (orange dashed line) and the high-frequency dielectric constant $\varepsilon_\infty$ (blue dashed line).

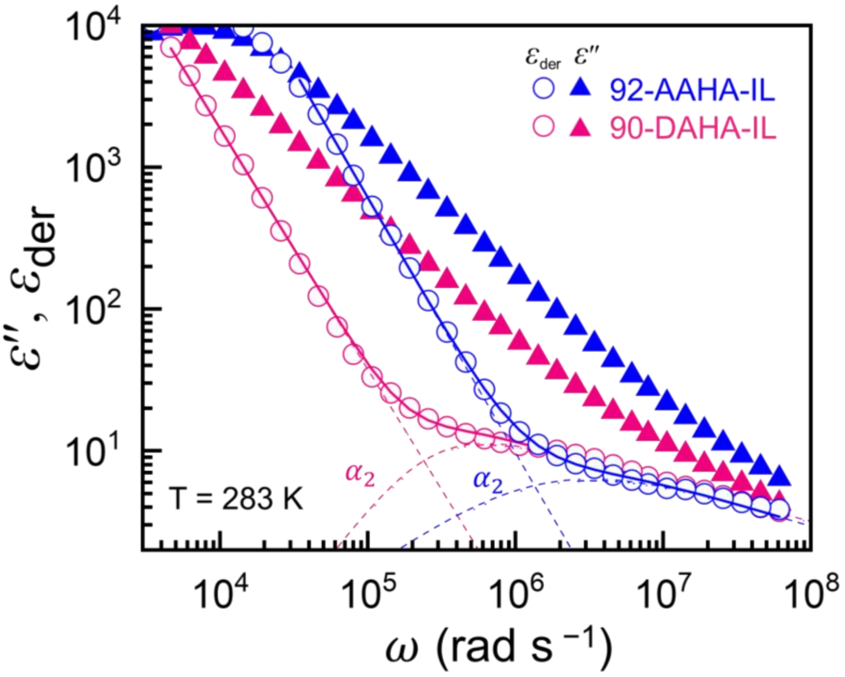


**Supplementary Fig. 16 Different $\alpha_2$ process between ionic- and non-ionic polymer-based ionoconductors.** Derivative (open circles) and dielectric loss (filled triangles) spectra at 283 K and corresponding fits using a sum (solid line) of a power law for electrode polarization (dashed line) and a derivative form of the Havriliak-Negami function for the $\alpha_2$ process (short dashed line).

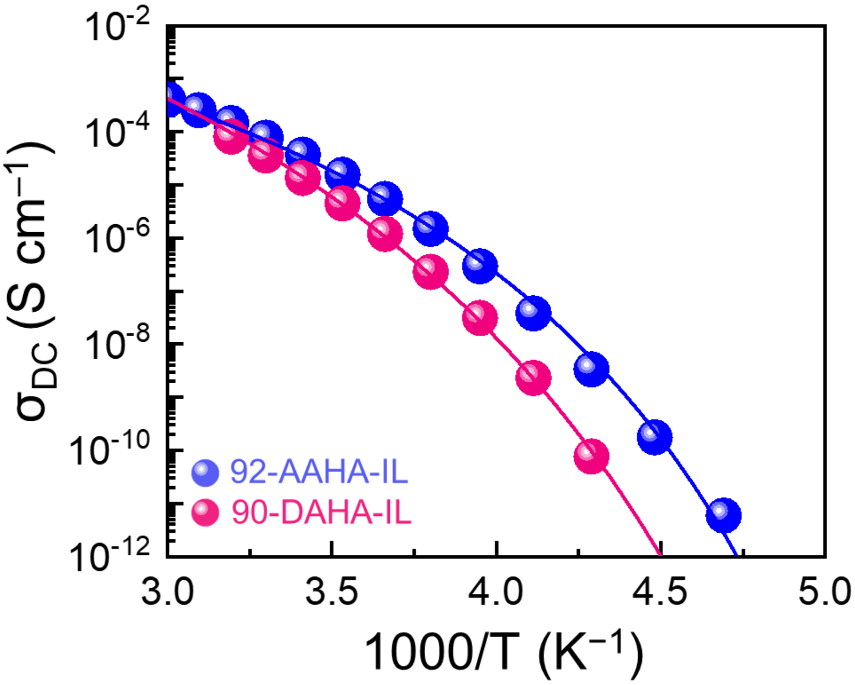


**Supplementary Fig. 17 Temperature dependent ionic conductivity.** Temperature dependence of ionic conductivity for 92-AAHA-IL and 90-DAHA-IL.

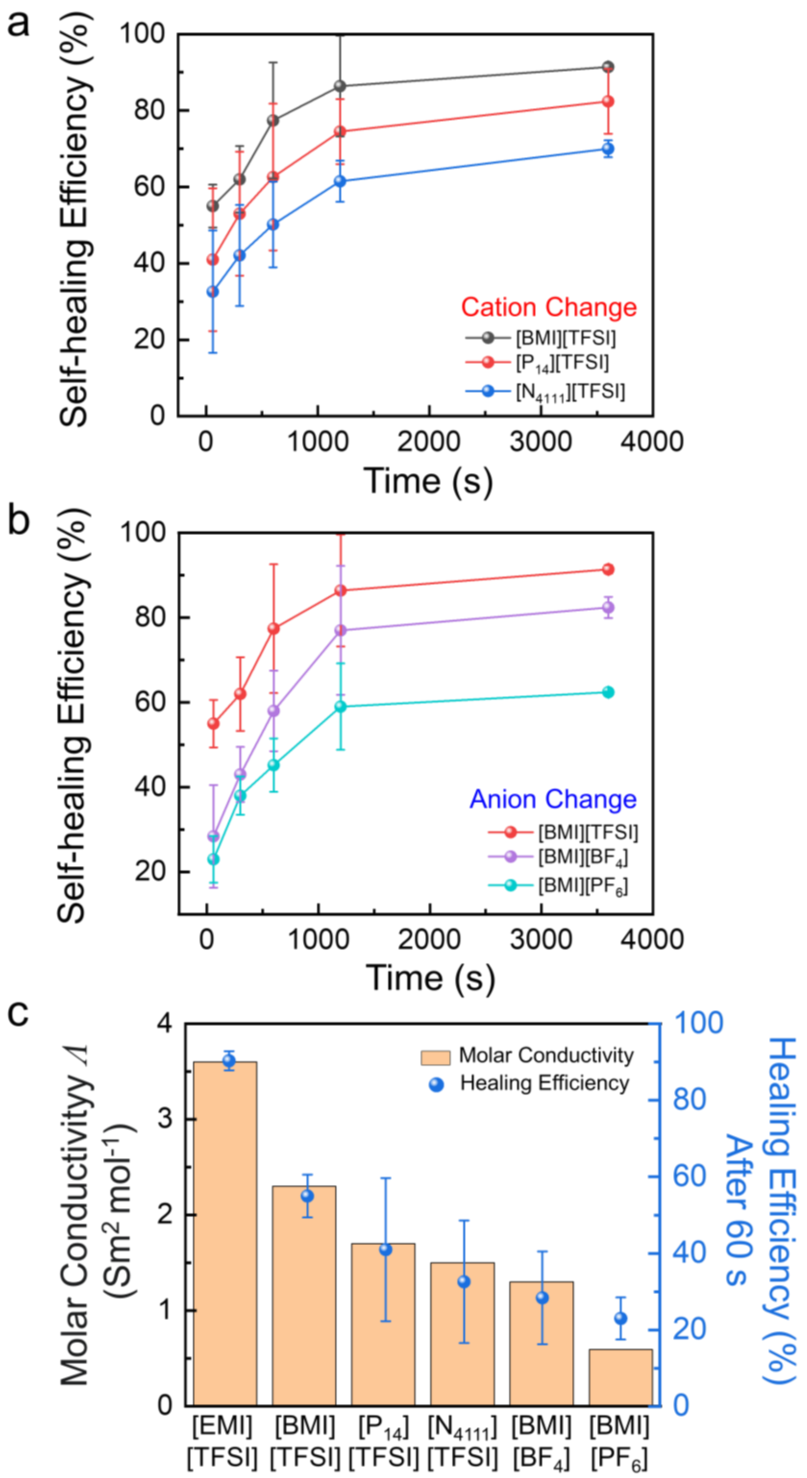


**Supplementary Fig. 18 Correlation between self-healing performance and type of ionic liquids.** Time-dependent self-healing efficiency of 92-AAHA-IL with selectively varied **a** cations and **b** anions of the ionic liquid. **c** Plots of molar conductivity ($\Lambda$) and healing efficiency of ionoconductors at a repair time of 60 s. The $\Lambda$ values were extracted from ref. 24. The self-healing processes of all ionoconductors were performed at 25 °C. Considering that a higher $\Lambda$ of ionic liquids indicates an easier ion–ion dissociation and ion movement, the superior performance of the [EMI][TFSI]-containing 92-AAHA-IL can be rationalized in terms of the highest $\Lambda$ of the ionic liquid additive. All error bars indicate standard deviation.

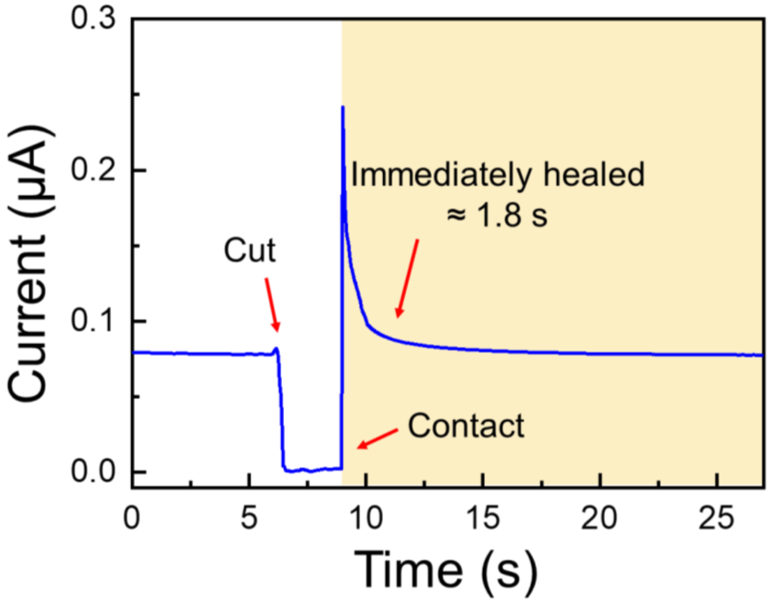


**Supplementary Fig. 19 Self-healing effect on electrical properties.** Self-healing of electrical properties of 92-AAHA-IL film. The flowing current was turned off when the film was cut in half, and was recovered immediately (~1.8 s) after reconnection.

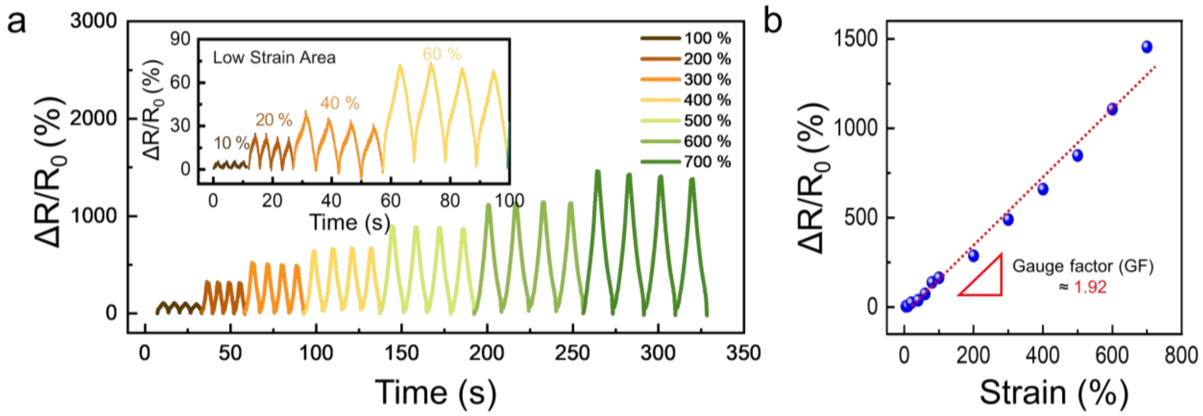


**Supplementary Fig. 20 Sensory performance of 92-AAHA-IL. a** Relative resistance variations ($\Delta R/R_0$) under applied strains up to 700% (inset: relative resistance changes at 10, 20, 40, and 60% strains). **b** Variations in $\Delta R/R_0$ over extended strain range, from which a GF value of ~1.92 was extracted. It should be noted that the ionoconductors alone can serve as ionic sensing platforms with no additional components. Accordingly, strain-sensing platforms can be readily fabricated by simply connecting electronic wires.

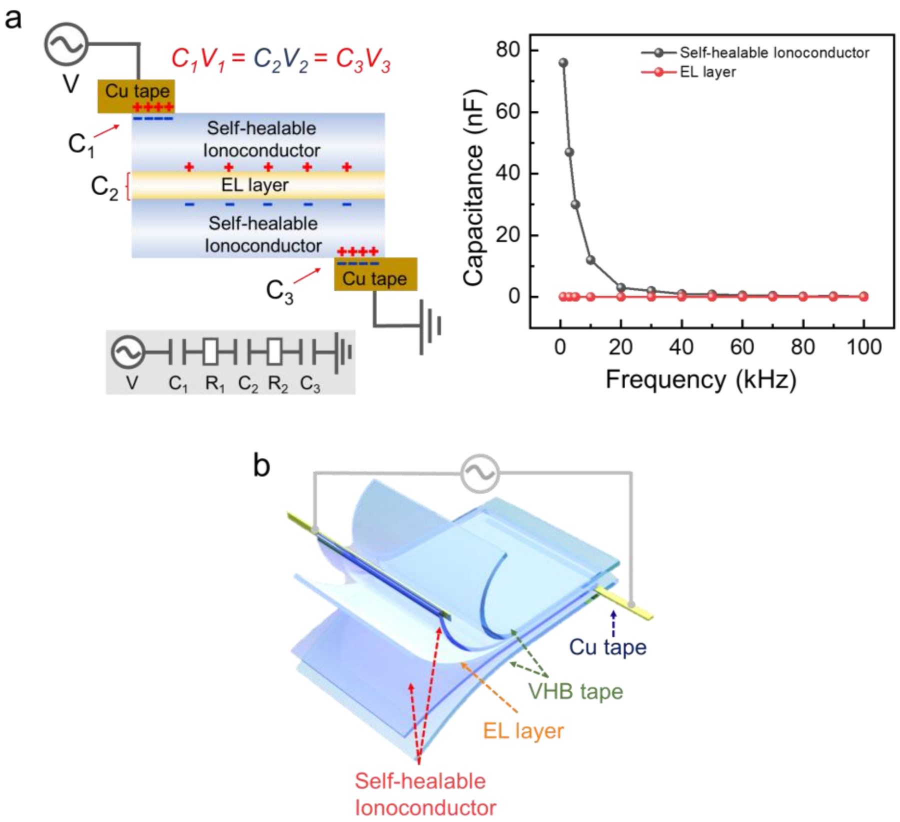


**Supplementary Fig. 21 Application of 92-AAHA-IL in AC electroluminescent displays (ACEDs). a** Schematic diagram (left) of ACED and capacitance variations (right) of each component (EL layer and 92-AAHA-IL-based ionoconductor). To construct electrical double layers (EDLs), Cu tape was attached to both sides of the ionoconductor. To avoid unnecessary interfacial electrochemical reactions, the EDL area ($A_{EDL}$) was a small portion of the total area of the ionoconductor ($A_{Ion}$). The EDL and dielectric layer acted as a series capacitor, following the equation: $Q = C_1V_1 = C_2V_2 = C_3V_3$, where $Q$ is the charge, while $C$ and $V$ represent the corresponding capacitance and voltage of each layer, respectively.[25,26] Accordingly, the EL layer had a relatively lower capacitance than the EDLs in ionoconductors in a lower frequency range (0.1 to 60 kHz), and the applied voltage was centered on the phosphor layer ($C_1/C_2$ or $C_3/C_2 \approx 1.8\times10^4$). **b** Schematic illustration of ACED. The EL device had a three-layer configuration where the EL phosphor layer was sandwiched between two symmetric ionoconductor layers.

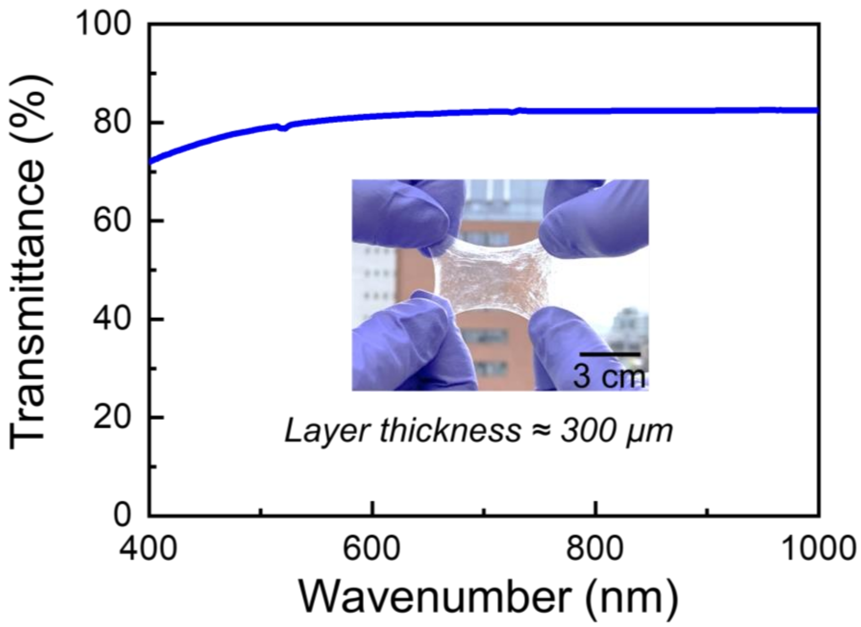


**Supplementary Fig. 22 Optical transparency of 92-AAHA-IL.** Transmittance spectrum of 300 μm-thick 92-AAHA-IL film at various wavenumbers. The overall transmittance values were higher than ~70 % at 400–1100 nm.

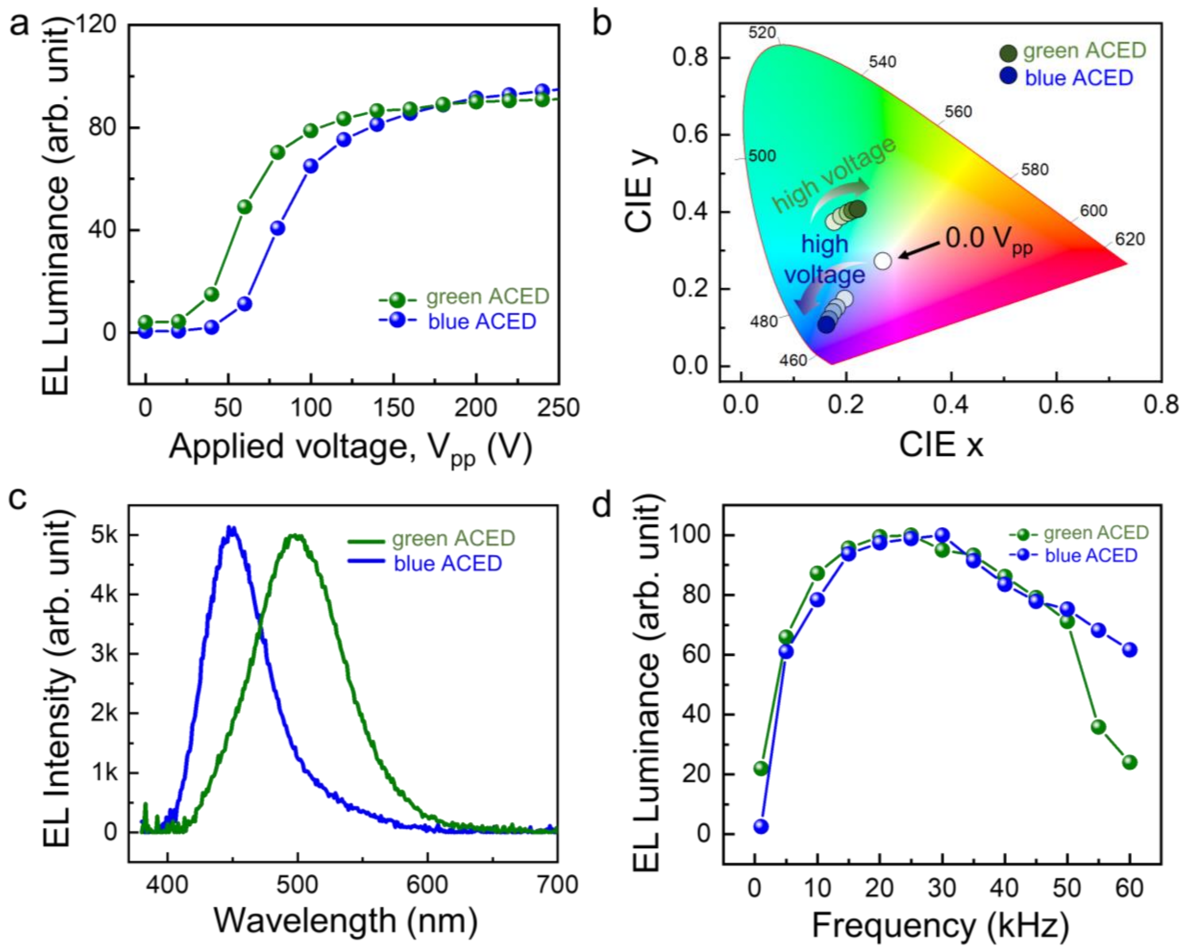


**Supplementary Fig. 23 Characteristics of AC electroluminescent displays (ACEDs). a** Luminance *versus* voltage characteristics and **b** CIE color coordinates of blue- and green-emitting ACED as a function of AC voltage at a frequency of 25 kHz. **c** EL emission spectra and **d** frequency dependence of EL luminance at an applied voltage of 200 $V_{pp}$. The magnitude of the emission intensity was linearly related to the driving voltage power from the turn-on voltage (~40 $V_{pp}$) to the saturation of both EL brightness above 200 $V_{pp}$ (Supplementary Fig. 23a). Each green- and blue-emitting luminophore successfully served as a deep color-brightening ACED upon applying various voltages (Supplementary Fig. 23b). The normalized EL spectra of the ACEDs confirmed the blue and green emission with maximum EL wavelengths at ~450 and ~500 nm (Supplementary Fig. 23c). The analysis of the frequency dependence showed that the EL intensity rapidly increased up to 25 kHz at the applied voltage of 200 $V_{pp}$ (Supplementary Fig. 23d). However, further frequency increases (above 30 kHz) resulted in a steady reduction in luminance, due to the decrease in the electron acceleration.[27] Therefore, the operating conditions of ACEDs were optimized at an applied voltage of 200 $V_{pp}$ and a frequency of 25 kHz to synchronize the green- and blue-emitting systems.

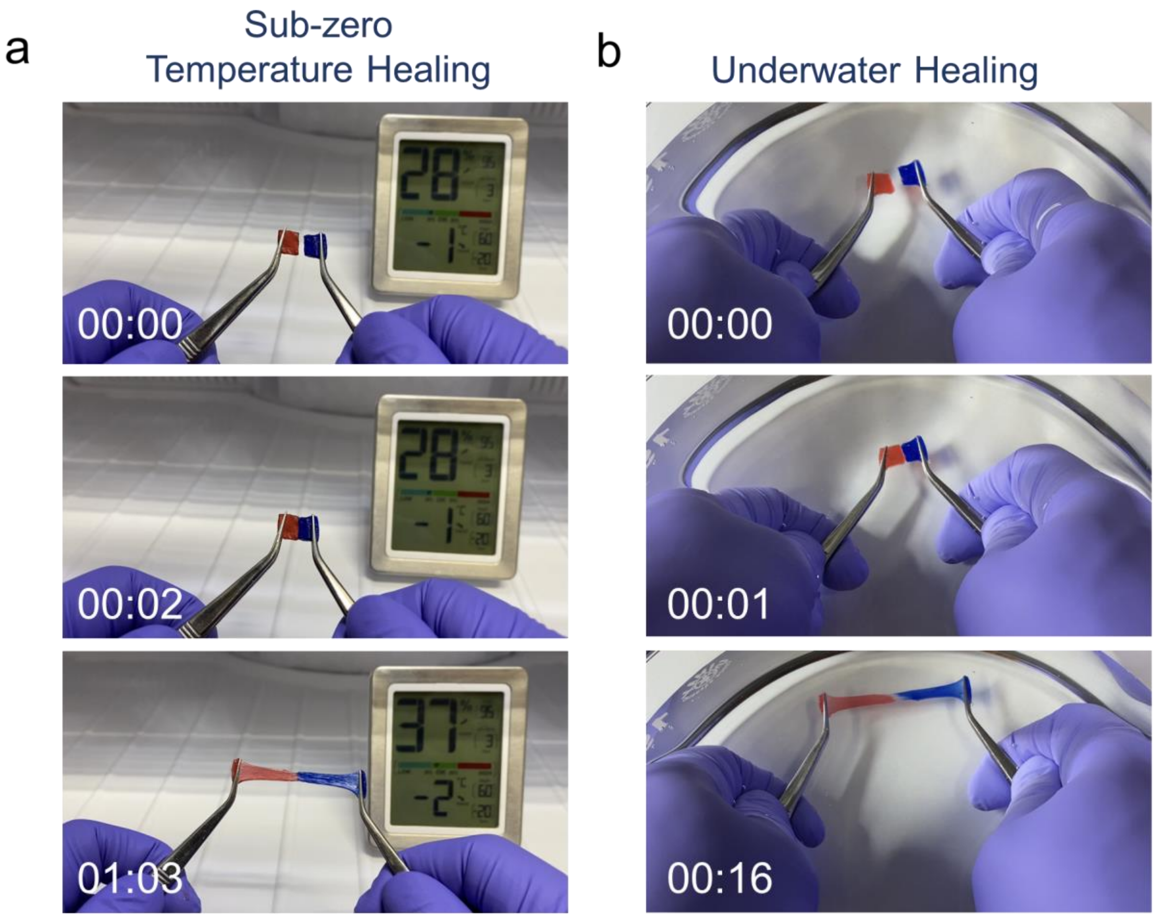


**Supplementary Fig. 24 Successful self-healing in various conditions.** Successful self-healing of 92-AAHA-IL in extreme environments: **a** sub-zero temperature and **b** underwater environment.

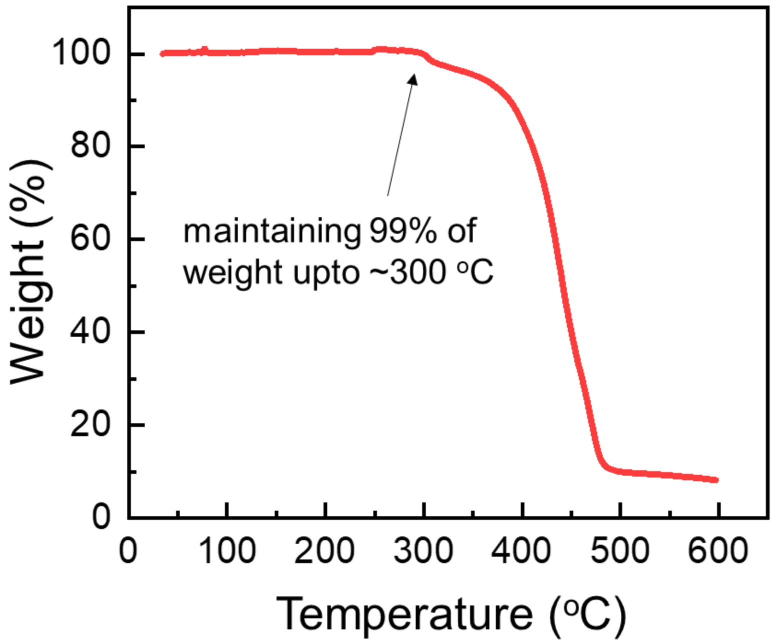


**Supplementary Fig. 25 Thermal stability of 92-AAHA-IL.** TGA thermogram of 92-AAHA-IL, indicating its high thermal stability up to ~300 $^{o}$C.

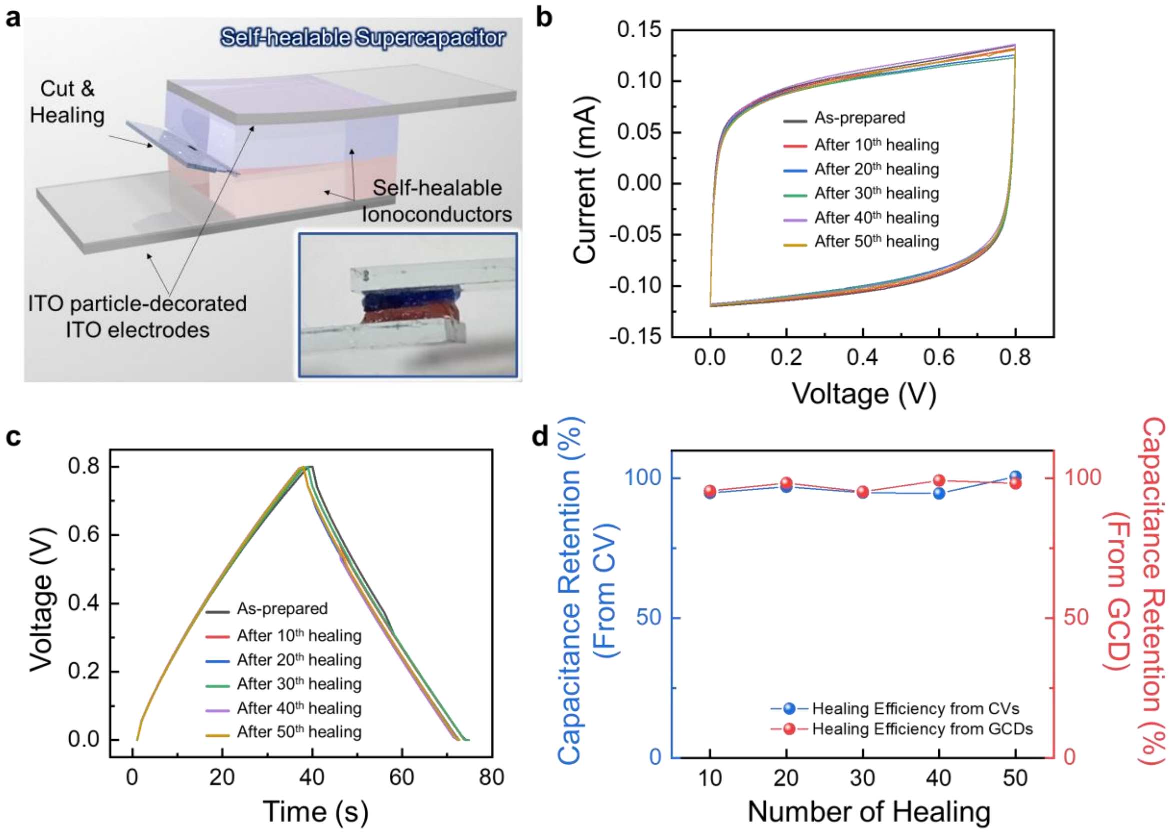


**Supplementary Fig. 26 Utilization of ultrafast self-healing performance of 92-AAHA-IL in healable supercapacitors.** **a** Schematic illustration of self-healable ionoconductor-based electrical double layer capacitor (EDLC). The inset displays a photograph of the actual device. **b** Cyclic voltammograms at a scan rate of 25 mV $s^{-1}$ during self-healing cycles. The areal capacitance ($C$) was calculated using the following equation:[28] $C = \frac{1}{\Delta V(dV/dt)} \int I\, dV$, where $dV/dt$ and $\int I\, dV$ are the voltage scan rate and the numerical integration of the current density in the half-cycle potential window ($\Delta V$), respectively. **c** Galvanostatic charge-discharge (GCD) profiles at a current density of 0.1 mA $cm^{-2}$. The $C$ values were extracted using the equation[29] $C = I\Delta t/S\Delta V$, where $I$, $\Delta t$, $S$, and $\Delta V$ represent the discharging current, discharging time, electrode area, and potential window excluding the *IR* voltage drop, respectively. **d** Variation in capacitance retention ($C/C_0$) as a function of number of healing cycles. A high $C/C_0$ was maintained irrespective of the number of self-healing cycles, demonstrating the practical applicability of 92-AAHA-IL in self-healable energy storage devices.

## Supplementary References


[1] Dai, X. et al. A mechanically strong, highly stable, thermoplastic, and self-healable supramolecular polymer hydrogel. *Adv. Mater.* **27**, 3566–3571 (2015).

[2] Jeon, I. et al. Extremely stretchable and fast self-healing hydrogels. *Adv. Mater.* **28**, 4678–4683 (2016).

[3] Gulyuz, U. et al. Self-healing poly(acrylic acid) hydrogels with shape memory behavior of high mechanical strength. *Macromolecules* **47**, 6889–6899 (2014).

[4] Zhang, Y. et al. Unique self-reinforcing and rapid self-healing polyampholyte hydrogels with a pH-induced shape memory effect. *Macromolecules* **54**, 5218–5228 (2021).

[5] Ihsan, A. B. et al. Self-healing behaviors of tough polyampholyte hydrogels. *Macromolecules* **49**, 4245−4252 (2016).

[6] Wang, L. et al. Tough, adhesive, self-healable, and transparent ionically conductive zwitterionic nanocomposite hydrogels as skin strain sensors. *ACS Appl. Mater. Interfaces* **11**, 3506–3515 (2019).

[7] Lei, J. et al. Facile fabrication of biocompatible gelatin-based self-healing hydrogels. *ACS Appl. Polym. Mater.* **1**, 1350−1358 (2019).

[8] Wu, M. et al. Ultra elastic, stretchable, self-healing conductive hydrogels with tunable optical properties for highly sensitive soft electronic sensors. *J. Mater. Chem. A* **8**, 24718–24733 (2020).

[9] Long, T. et al. Salt-mediated polyampholyte hydrogels with high mechanical strength, excellent self-healing property, and satisfactory electrical conductivity. *Adv. Funct. Mater.* **28**, 1804416 (2018).

[10] Chen, X. et al. Extremely tough, puncture-resistant, transparent, and photoluminescent polyurethane elastomers for crack self-diagnose and healing tracking. *ACS Appl. Mater. Interfaces* **12**, 30847−30855 (2020).

[11] Li, J. et al. Seawater-assisted self-healing of catechol polymers via hydrogen bonding and coordination interactions. *ACS Appl. Mater. Interfaces* **8**, 19047−19053 (2016).

[12] Li, R. et al. Autonomous self-healing, antifreezing, and transparent conductive elastomers. *Chem. mater*. **32**, 874−881 (2020).

[13] Li, X. et al. Self-healing polyurethane elastomers based on a disulfide bond by digital light processing 3D printing. *ACS Macro Lett.* **8**, 1511−1516 (2019).

[14] Ying, W. B. et al. A biologically muscle-inspired polyurethane with super-tough, thermal reparable and self-healing capabilities for stretchable electronics. *Adv. Funct. Mater.* **31**, 2009869 (2021).

[15] Xu, W. M. et al. Sunlight driven self-healing, reshaping and recycling of a robust, transparent and yellowing-resistant polymer. *J. Mater. Chem. A* **4**, 10683–10690 (2016).

[16] Li, C.-H. et al. A highly stretchable autonomous self-healing elastomer. *Nat. Chem.* **8**, 618–624 (2016).

[17] Zhu, M. et al. Long-lasting sustainable self-healing ion gel with triple-network by trigger-free dynamic hydrogen bonds and ion bonds. *ACS Sustain. Chem. Eng.* **6**, 17087−17098 (2018).

[18] Tamate, R. et al. Self-healing micellar ion gels based on multiple hydrogen bonding. *Adv. Mater.* **30**, 1802792 (2018).

[19] Saruwatari, A. et al. Photohealable ion gels based on the reversible dimerisation of anthracene. *Chem. Commun.* **54**, 13371−13374 (2018).

[20] Ueki, T. et al. Thermally reversible ion gels with photohealing properties based on triblock copolymer self-assembly. *Macromolecules* **48**, 5928−5933 (2015).

[21] Cao, Y. et al. A transparent, self-healing, highly stretchable ionic conductor. *Adv. Mater.* **29**, 1605099 (2017).

[22] Cao, Y. et al. Self-healing electronic skins for aquatic environments. *Nat. Electron.* **2**, 75–82 (2019).

[23] Zhao, D. et al. A dynamic gel with reversible and tunable topological networks and performances. *Matter* **2**, 390−403 (2020).

[24] Tokuda, H. et al. How ionic are room-temperature ionic liquids? an indicator of the physicochemical properties. *J. Phys. Chem. B* **110**, 19593–19600 (2006).

[25] Wang, J. et al. Extremely stretchable electroluminescent devices with ionic conductors. *Adv. Mater.* **28**, 4490–4496 (2016).

[26] Xuan, H. D. et al. Super stretchable and durable electroluminescent devices based on double-network ionogels. *Adv. Mater.* **33**, 2008849 (2021).

[27] Liang, G. et al. Self-healable electroluminescent devices. *Light-Sci. Appl.* **7**, 102 (2018).

[28] Allagui, A. et al. Reevaluation of Performance of Electric Double-layer Capacitors from Constant-current Charge/Discharge and Cyclic Voltammetry. *Sci. Rep.* ***6***, 38568 (2016).

[29] Kim, S. Y. et al. Reliable, high-performance electrochromic supercapacitors based on metal-doped nickel oxide. *ACS Appl. Mater. Interfaces* **12**, 51978-51986 (2020).
