# Supplementary material for: Ion-cluster-mediated ultrafast self-healable ionoconductors for reconfigurable electronics
Source: Nat Commun. 2022 Jun 30;13:3769. doi: 10.1038/s41467-022-31553-4 (PMC9247092; doi:10.1038/s41467-022-31553-4)
Supplement: Supplementary file 3 — Description of Additional Supplementary Files [file 41467_2022_31553_MOESM3_ESM.pdf]

Description of Additional Supplementary Files

File Name: Supplementary Movie 1
Description: This movie shows the superior self-healing performance of 92-AAHA-IL demonstrated by blocking picometer-sized nitrogen gas molecules after self-healing.

File Name: Supplementary Movie 2
Description: This movie demonstrates the ultra-fast self-healing capacity of the 92-AAHA-IL-based ionoconductor.

File Name: Supplementary Movie 3
Description: This movie shows the facile fabrication of a multi-color emitting ACED by simply connecting two ACEDs.

File Name: Supplementary Movie 4
Description: This movie illustrates the application of the self-healing properties in a pixelated ACED. Square-shaped ACEDs with green- and blue-emissive layers (10 mm length × 10 mm width × 700 μm thickness) were connected to form a 3×3 pixelated ACED. After self-healing, the pixelated ACED successfully operated under various deformations. This highlights the great potential of the present systems in reconfigurable displays.

File Name: Supplementary Movie 5
Description: This movie demonstrates the self-healing capacity of 92-AAHA-IL in sub-zero temperature conditions.

File Name: Supplementary Movie 6
Description: This movie demonstrates the self-healing capacity of 92-AAHA-IL underwater environments.
